# Development of a Clean-Label Meat-Free Alternative to Deli Ham

**DOI:** 10.3390/foods14142416

**Published:** 2025-07-08

**Authors:** Lisiane Carvalho, Beatriz Caetano, Capucine Godinot, Norton Komora, Adriana Ferreira, Célia Rocha, Bruna Barbosa, Anabela Raymundo, Isabel Sousa

**Affiliations:** 1 LEAF—Linking Landscape, Environment, Agriculture and Food, Instituto Superior de Agronomia, Universidade de Lisboa, Tapada da Ajuda, 1349-017 Lisboa, Portugal; beatriz.caetano@outlook.com (B.C.); capucine.godinot@gmail.com (C.G.); nortonsnk@hotmail.com (N.K.); anabraymundo@isa.ulisboa.pt (A.R.); 2 R&D Departament, Primor Charcutaria-Prima, S.A., Gavião, 4760-003 Vila Nova de Famalicão, Portugal; adriana.ferreira@primor.pt; 3 Sense Test, Sociedade de Estudos de Análise Sensorial a Produtos Alimentares Lda., 4400-345 Vila Nova de Gaia, Portugal; celia.rocha@sensetest.pt (C.R.); bruna.barbosa@sensetest.pt (B.B.); 4 GreenUPorto—Sustainable Agrifood Production Research Centre/Inov4Agro, DGAOT, Faculty of Sciences, University of Porto, Campus de Vairão, 4485-646 Vila do Conde, Portugal

**Keywords:** additive-free, clean label, deli ham, meat-free

## Abstract

Reducing meat consumption is a key strategy to mitigate environmental impact, lower the incidence of diet-related diseases, and promote sustainable food production. In response, the plant-based food market has grown significantly, motivated by demand for meat-like products. This study aimed to develop a meatless alternative to deli ham (MAD) free of chemical additives, adhering to clean label principles. A commercially available MAD product (Target MAD) was used as a benchmark. Based on its analysis, clean-label laboratory (Optimized CL formulation) and pilot-scale (CL MAD) prototypes were developed. These were evaluated for texture, rheology, color, sensory attributes, and physicochemical properties. The CL MAD demonstrated similar firmness to the Target MAD, while being 17% more cohesive and 50% less adhesive. Its mechanical spectra showed typical weak gel behavior, with G′ higher than G″. Color analysis indicated that the CL MAD was darker and less pink than the Target MAD. Nutritionally, it provided higher protein and lower fat content. Overall, this study successfully developed a clean-label meat-free alternative to deli ham that matches commercial textural standards while offering improved nutritional quality and eliminating chemical additives, meeting growing consumer demand for healthier and more sustainable foods.

## 1. Introduction

The practice of vegetarianism is increasing worldwide, due to health-related risks, religious and ethical reasons, among others, raising the value of the plant-based food market [1,2]. The global market for meat analogue products is forecast to reach 3.5 billion dollars by 2026, with an annual growth rate of 12%, with the largest market share held by Europe (51.5%) [3]. Along with this growth, there is increasing demand for convenient, meat-like, high-protein foods, leading to the development of meat analogues [4].

According to a recent trend report by EIT Food (European Institute of Innovation and Technology), one of the top five European food trends in 2024 was the demand for more consumer information and personalized nutrition. Aligned with the European Commission’s strategy for research and innovation in protein diversification, an increase in innovation for meat alternatives is expected, with emphasis on personalization, convenience, and taste [5].

Despite the market’s rapid growth, concerns remain regarding the nutritional value and healthfulness of commercial meat substitutes [6]. These products are expected to emulate the texture, appearance, taste, and nutritional value of traditional meat, often requiring equivalent technological processes [4].

However, plant-based formulations still face significant challenges, including poor fibrous structure, chewiness, and juiciness. Replicating meat-like flavor and achieving characteristic red or pink coloration also remain obstacles. Moreover, the lower essential amino acid content in plant proteins poses a nutritional challenge [7]. In addition to this, a growing number of health-conscious and informed consumers are seeking more natural, minimally processed food products [6,8].

Among plant-based meat analogues, cold cuts (e.g., deli ham) represent a specific category of processed meats where consumer expectations for authentic sensory and technological characteristics are especially high. Replacing the typical animal-based matrix in deli meats is particularly challenging due to the functional roles of conventional additives in maintaining texture, water retention, sliceability, and flavor. Traditional hydrocolloids such as phosphates (E450–E452), carrageenan (E407), and konjac gum (E425) are widely used in processed meat products for their emulsifying capacity, water-binding ability, and role in enhancing texture and oxidative stability [9,10].

However, in recent years, the industry has experienced a shift toward less refined and more natural food solutions, leading to the emergence of the “Clean Label” concept, a top food trend widely embraced by consumers and manufacturers [11,12,13,14,15]. Clean-label products are generally perceived as ‘natural’, ‘organic’, and/or ‘free from additives’ and devoid of ingredients that could be perceived as synthetic or overly processed. These products typically feature short and familiar ingredient lists [16].

The clean label concept lacks a formal legal definition, creating ambiguities in product classification, particularly concerning terms such as “natural” and “artificial.” Consumers increasingly reject synthetic additives and complex names in favor of simpler ingredient lists, pressuring manufacturers to find natural alternatives for functional ingredients such as flavorings, colorants, preservatives, and other artificial additives [17,18].

Technological challenges arise when attempting to meet clean label standards while ensuring product stability, sensory quality, and safety. In clean-label cold cut substitutes, promising natural alternatives have been explored. Ingredients such as psyllium, pre-gelatinized banana starch, inulin, fructooligosaccharides (FOSs), and flours from cassava, rice, and quinoa have shown potential for mimicking the functionalities of conventional hydrocolloids [19,20,21,22,23,24,25,26,27,28,29].

Innovation in food processing, including extrusion, shear cell technology, and freeze structuring, has helped improve the texture, flavor, and nutritional properties of plant-based cold cuts. Plant sources are also being investigated for their functional and nutritional profiles [30]. Nonetheless, the formulation of clean-label deli-style products without the use of traditional additives still represents a significant technological and sensory challenge.

Therefore, the objective of this study was to develop a clean-label meatless alternative to deli ham (CL MAD) using natural, minimally processed ingredients, in accordance with clean label principles.

## 2. Materials and Methods

**Figure 1:**
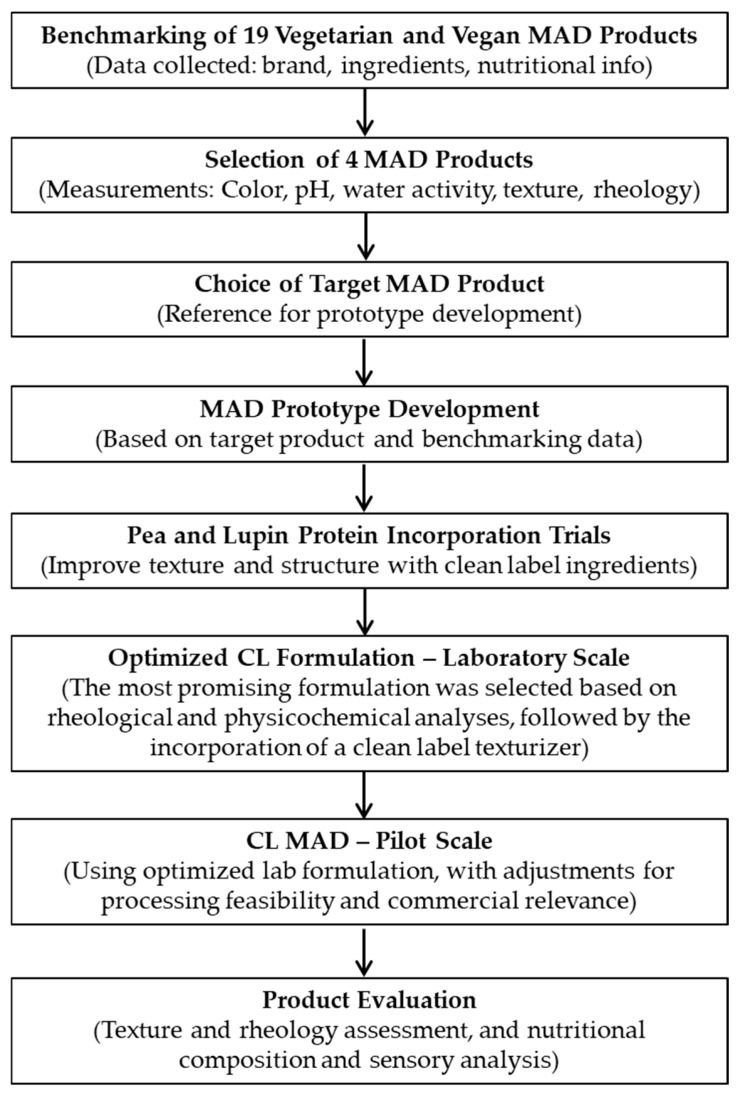
Workflow diagram illustrating the sequential stages of the CL MAD product development, from market benchmarking through laboratory formulation, scale-up, and final product evaluation.

To provide a clearer overview of the sequential steps involved in the CL MAD development, Figure 1 presents a workflow diagram summarizing the entire process. Before starting the development of a clean-label meatless alternative to deli ham (CL MAD), a benchmarking with 19 vegetarian and vegan MAD products, both from national and international brands, was conducted to understand the MAD products already on the market and to record their characteristics, brand, list of ingredients, and nutritional information. From this initial selection, 10 products were chosen for further analysis, and their relevant properties, including color, pH, water activity, texture, and rheology, were measured. Based on the outcomes of these analyses, four commercial deli ham analogues were selected based on their relevant attributes, one of which was chosen as the target product.

The MAD prototype formulation was developed at pilot scale. Additionally, pea protein concentrate and lupin protein isolate incorporation trials were conducted to improve the formula at a laboratory scale. The following was intended to replace the incorporated food rheology improvers, responsible for the thickening and gelling properties, but as clean-label alternatives. The formula optimization was carried out using the most successful clean-label natural fiber texturizer. Color, pH, moisture and ash content, water activity, texture, and rheology characteristics were accessed in the final products. Once the formulation had been optimized at a laboratory scale, a scaling-up test was conducted. This stage was performed at a pilot-scale line, closer to the industrial scale, applying the optimized formulation. Finally, a sensory analysis was carried out, to understand the acceptance of the pilot product, as well as analysis of nutritional composition, texture and rheology. All analyses were performed in at least triplicate.

### 2.1. MAD Formulations

After weighing the ingredients (water, ice, sunflower oil, egg white powder, potato starch, hydrocolloid mixture, dextrose, spices, and colorant), homogenization was carried out at pilot scale (MAD prototype) using a pilot bowl cutter CM-41 (Mainca, Barcelona, Spain) (maximum capacity of 25 kg), where ice is incorporated so that the mixture does not exceed 6 °C, to avoid hindering the gelling process. Once the ingredients had been added, all at once, the equipment lids were closed and the speed increased to obtain the emulsion mixture. This mixture was subsequently transferred into a VF 608 plus vacuum filler (Handtmann, Biberach, Germany) coupled to a semi-automatic double-clipper PDC 600 (Poly Clip system, Hattersheim am Main, Germany), which filled the mixture into a polyamide/polyethylene (PA/PE) plastic casing, vacuum-sealed by clipping.

For the laboratory scale (LS MAD), the same ingredients used at pilot scale were used, and two more formulations were evaluated, where 5% of potato starch was replaced by 5% lupin protein isolate (prototype 5% LPI) and by 5% pea protein concentrate (prototype 5% PPC). The mixture was homogenized in a Thermomix TM31 (Bimby, Vorwerk, Wuppertal, Germany) and the filling into the plastic casing and sealing were performed manually.

The MADs were steam-cooked (Verinox, Altopiano della Vigolana, Italy) (pilot scale) or cooked in a water bath (laboratory scale) at 77 °C until the thermal center reached 70 °C. After that, the products were placed in a cold room at 4 °C for at least four days to stabilize.

After defining the best formulation, the replacement of added E-number hydrocolloids (phosphates (E450–E452), carrageenan (E407), and konjac gum (E425)) with different clean-label texturizers (psyllium, pre-gelatinized banana starch, inulin, fructooligosaccharides, and flours from cassava, rice, and quinoa) was evaluated, and one formulation was developed at a laboratory scale (Optimized CL formulation). To analyze if the scale-up influenced the product’s characteristics, a pilot-scale (CL MAD) trial was conducted. Since the pilot-scale equipment turns the mixture denser and more compact, the CL MAD formulation underwent some final adjustments, such as changing the order of ingredient incorporation to optimize the mixing step, and flavoring ingredients and frozen vegetables (broccoli and baby carrots) were added. The water content was also reduced, since 10% of the formulation consisted of frozen vegetables, and the colorant content was reduced as well.

### 2.2. Texture Analysis

A Texture Profile Analysis (TPA) was carried out using a Texturometer TA-XT plus (Stable Micro Systems, Surrey, United Kingdom) with a 5 kg load cell in a temperature-controlled room at 20 ± 1 °C [31]. Ten repetitions were analyzed for each sample. The TPA was performed using a cylindrical probe of 10 mm diameter, 35 height. The samples were prepared placing 5 to 10 slices in a small pile with a height of 1 ± 0.2 cm. The TPA test was performed in penetration mode at a test speed of 1 mm/s, with a 5 s waiting time, and a penetration distance of 5 mm. Force (N) versus time (s) curves were obtained, and the main representative parameters were determined, including firmness (N), adhesiveness (-N/s), and cohesiveness.

### 2.3. Rheology Measurements

A controlled-stress rheometer (Haake Mars III, Thermo Fischer Scientific, Waltham, MA, USA) was used, connected to an air compression system (Eheim professional 3) and a Peltier temperature control unit [32]. A serrated parallel plate system with a 20 mm diameter (PP20) was used, and a slice from each sample was analyzed. Prior to the frequency sweep, a stress sweep test was conducted, at 1 Hz, to identify the linear viscoelastic region (LVR). The viscoelastic characteristics were evaluated though frequency sweep tests, within the LVR, across an angular frequency range of 0.01 to 100 Hz. All tests were conducted at a temperature of 10 ± 0.5 °C, close to the consumption temperature of this kind of product, in a temperature-controlled room at 20 ± 1 °C. At least three replications were analyzed. The mechanical spectrum (i.e., G′ and G″ as a function of frequency) and the loss tangent (tan δ = G″/G′) were obtained. The plateau modulus (G_N_^0^) was estimated as the value of G′ obtained for the minimum value of the loss tangent, as described in the literature [32].

### 2.4. Color Analysis

The color determination was carried out using the CIELAB scale, with illuminant C, by direct reading of the samples using a Chroma Meter CR 400 Series Konica Minolta (Osaka, Japan), calibrated by a standard white plate (L* = 94.68; a* = −0.52; b* = 3.47). All measurements were performed on 6 distinct areas of a slice under the same conditions. To compare the differences between two samples, the total color difference (ΔE) was calculated according to the following Equation (1) [33]:

(1) $$∆E=\sqrt{{\left(L_{i}^{*}-L_{0}^{*}\right)}^{2}+{\left(a_{i}^{*}-a_{0}^{*}\right)}^{2}+{\left(b_{i}^{*}-b_{0}^{*}\right)}^{2}}$$

where *i* indicates the analyzed sample, and 0 represents the targeted sample. When comparing the samples, it is considered that the observer detects distinct colors if the Δ*E* value is more than 5 [34].

### 2.5. pH Analysis

The pH measurement was estimated using a pH-meter BASIC 20+ (Crison Instruments, Barcelona, Spain) coupled to a 50 11 T pH electrode (HACH, Loveland, CO, USA) and determined in triplicate, measuring different areas of a pile of slices of 2 ± 0.3 cm height.

### 2.6. Water Activity Analysis

The water activity (a_w_) was evaluated using the HygroPalm HP23-AW-A (Rotronic, Bassersdorf, Switzerland) apparatus coupled with a GH refrigerated water bath (Haake, Fisons, Karlsruhe, Germany). The materials were minced and analyzed in triplicate, in a temperature-controlled room at 20 ± 1 °C.

### 2.7. Nutritional Composition

Nutritional composition was analyzed using samples previously minced, and the measurements were performed in triplicate. The moisture content was measured as described by AACC International Method 44-15.02 [35] and determined gravimetrically at 100–105 °C (Binder, ED115, Tuttlingen, Germany) until a constant weight was obtained. The total ash content was measured by incineration for 24 h, at 550 °C in a muffle furnace (SNOL, 13/1100, Utena, Lithuania) according the Portuguese standard NP 1615 [36].

The total lipids content was determined as described in the Portuguese standard NP 1224 [37]. The minced sample was placed in a paper bag inside a Soxhlet extraction apparatus and extracted by n-hexane. Then, the solvent was evaporated at a rotary evaporator. Then, the flask was dried in an oven overnight at 60 °C. The total lipids content was calculated by the weight difference and expressed in dry matter.

The protein content was estimated through an NDA 702 Dumas Nitrogen Analyzer (VELP scientifica, Usmate Velate, Italy), powered by DUMASoft software version 6.1.0. The nitrogen was measured according to the Dumas combustion method, subjecting the sample to a combustion at a temperature higher than 1000 °C to obtain elemental compounds. These compounds pass through a reduction furnace, where nitrogen oxides (Nox) compounds are transformed into molecular nitrogen, which is detected by a thermal conductivity detector, TCD LoGas, which sends the data to DumaSoft, applying a conversion factor of 6.25 to transform the results into protein [31].

The mineral content was quantified through the Inductively Coupled Plasma Optical Emission Spectrometry (ICP-OES) Thermo Scientific ICAP Series 7000 (Thermo Fisher Scientific, Waltham, MA, USA) [38]. For this measurement, approximately 0.2 g of minced sample were introduced in a 50 mL plastic vial and digested with a concentrated acid mixture of 8 mL of hydrochloric acid (HCl) and 2 mL of nitric acid (HNO_3_). The digestion process was carried out at 105 °C for 90 min using an open-vessel system. Then, the resulting solution was diluted to 50 mL with distilled water and agitated slowly. For the mineral determination, 10 mL of the prepared clear solution was analyzed.

The carbohydrate content was calculated by subtracting the sum of the other components (protein, lipid, ash, and moisture) from 100. The energy value of the samples was obtained by calculation, using the average energy values of each nutrient: 4 kcal/g carbohydrates, 4 kcal/g protein, and 9 kcal/g lipids according to Regulation (UE) n.^o^ 1169/2011 [39], combined with the results obtained earlier regarding the centesimal composition.

### 2.8. Sensory Analysis

To evaluate the relevant factors in the choice of a MAD, consumers’ perception of the global acceptance of six different samples, including the MAD prototype, CL MAD, and four commercial deli ham analogues, one of which was the commercial Target MAD, was measured. The sensory evaluation was performed by 65 participants and carried out in individual sensory booths at Sense Test (a sensory evaluation and consumer testing company in Vila Nova de Gaia, Portugal). The sensory evaluation laboratory was equipped in accordance with ISO 8589:2007—Sensory analysis—General guidance for the design of test rooms [40], and the appropriate protocols for protecting the rights and privacy of all participants were applied during the execution of the research. Volunteers received informed consent forms, in line with the ethical guidelines of the local human experimentation committee and the World Medical Association’s Code of Ethics (Declaration of Helsinki, 1975, revised in 2013).

For each sample, overall liking was assessed using a 9-point scale, ranging from 1 (dislike extremely) to 9 (like extremely) [41]. To minimize potential carry-over effects, each participant received the set of samples following a monadic sequential order of presentation, with a balanced presentation order [42].

In a second session, the samples were once again delivered simultaneously to the participants, and they were invited to perform a projective mapping exercise [43]. In this approach, the participants were invited to evaluate the overall characteristics of the samples and position them on the map, following similarity criteria. Once the positioning map was finalized, the consumers were instructed to describe the sensory characteristics of each sample to justify the positioning defined, using the approach of Ultra Flash Profiling [44]. The projective mapping exercise was conducted on a computer system with a screen resolution of 1920 × 1080 (100%).

### 2.9. Statistical Analysis

The results were statistically analyzed using an analysis of variance (ANOVA), and post-hoc tests, a *t*-test for two samples and Tukey’s test for three samples were carried out. For sensory evaluation, simple descriptive statistics of overall liking data were performed, and two nonparametric tests, the Friedman and Wilcoxon tests, were applied [45]. A Multiple Factor Analysis (MFA) was used to perform a consensus analysis comparing the individual product position on the projective mapping task, the ultra-flash profile data, and overall liking, identifying whether a particular attribute was associated with a given sample [46] and determining the liking drivers, using XLSTAT 2024 (Lumivero PF, Denver, CO, USA). All statistical tests were applied at a 95% confidence level (*p* < 0.05).

## 3. Results and Discussion

### 3.1. Market Research and Preliminary Physicochemical Analysis

After carrying out the benchmarking study on commercialized meat-free alternatives to deli ham (MADs), including their relevant properties, it was possible to identify that the ingredients used in the formulations were water, vegetable oil (canola, sunflower, coconut, and olive oil), vegetable protein isolates (pea, soy, and wheat) and egg white protein powder, polysaccharides (mainly xanthan gum, carrageenan, and locust bean gum), yeast extract, coloring agents (extracts from beetroot, aronia berries, horseradish, blackcurrant, tomato lycopene and paprika), spices (onion, garlic, pepper, leek powder, natural smoke, and chicken flavoring), salt, and sugars (maltodextrin, dextrose, and glucose syrup).

The analysis of the properties of commercial MAD, selected as the target (Target MAD), and the MAD prototype (Table 1) provided a general overview of the characteristics of these products. According to the results, the MAD prototype showed a more yellowish tone (*p* < 0.05) than the Target MAD. In addition, for the color coordinates a* and L*, no significant differences (*p* > 0.05) between the samples were found. Regarding the color difference, the ΔE value was 5.07, indicating that the color difference was almost imperceptible to the human eye [34]. According to Ryu et al. [47], plant-based meat analogues can replicate the color of meat by incorporating plant pigments, such as beetroot extract, caramel pigment, carotene, tomato paste, and pomegranate extract. A mixture of these is generally used, as the incorporation of a single pigment may inadequately replicate the desired color range, both externally and internally.

Regarding the other parameters, both samples exhibit significant differences (*p* < 0.05) in a_w_, firmness, and cohesiveness, with the Target MAD showing higher values than the MAD prototype. Additionally, the Target MAD had lower pH and ash values. However, no significant differences (*p* > 0.05) were found for the viscoelastic rheology parameters, moisture content, and adhesiveness values, which is a positive outcome.

The lower a_w_ observed for the MAD prototype may be due to the presence of starch, since according to Farooq and Boye [48], the presence of proteins together with starch and fiber causes molecular interactions that favor water absorption and gel formation upon heating and subsequent cooling.

Another important parameter is pH, with both samples presenting values around 6, which likely contributes to protein solubilization and influences the emulsification ability, foaming, and gelling properties of the products. Ladjal and Chibane [49] verified, for example, that for legume flours, protein solubility gradually increased below pH 3.0 and above pH 5.0, reaching a maximum at high alkaline pH values (≈pH 8) and low acidic pH values (≈pH 2).

To meet consumer expectations regarding meat-like attributes, it is essential to address technological challenges related to alternative proteins, particularly in replicating texture. For instance, the globular proteins found in plants lack the ability to form the fibrous structures characteristic of muscle tissue, as achieved by myosin and actomyosin, making it difficult to reproduce the complex and subtle sensory properties of conventional meat products. The degree of proteins texturing can be improved through two approaches: the selection of raw materials and the processing methods [7].

### 3.2. Development of MAD Prototype in a Laboratory and Pilot Scale

Taking into account the formulation process and the formula composition benchmarked, the MAD prototype (with 5% potato starch) was reproduced at a laboratory scale (LS MAD). Simultaneously, 5% of the potato starch in the LS MAD formulation was replaced with 5% lupin protein isolate (prototype 5% LPI), with a protein content of 90.9% ± 0.06, and with 5% pea protein concentrate (prototype 5% PPC), with a protein content of 55.2% ± 0.16.

**Figure 2:**
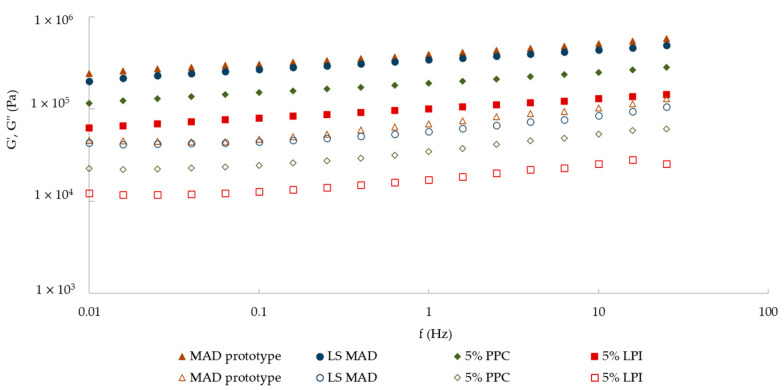
Modulus of conservation (elastic), G′ (solid symbols), and dissipation (viscous) G″ (open symbols) of the MAD prototype, LS MAD, 5% PPC, and 5% LPI.

Figure 2 shows the mechanical spectra of these formulations (LS MAD, 5% PPC, and 5% LPI) in comparison with the MAD prototype. All four samples revealed similar mechanical spectra, with the elastic modulus (G′) higher than the viscous modulus (G″) by nearly a decade, demonstrating a predominantly elastic behavior characteristic of a very structured weak gel, since all mechanical spectra showed a slight frequency dependence. This pattern was expected, as all the formulations were similar, and the ingredients were incorporated within a specific order and pace.

The mechanical spectra of the prototypes 5% PPC and 5% LPI evidenced lower G′ and G″ results compared to LS MAD and the MAD prototype, showing a weaker structure. The 5% LPI prototype showed significant (*p* < 0.05) lower results than the 5% PPC, revealing that LPI forms weaker gels. Batista et al. [50] showed similar results when studying the gelling ability of vegetable proteins. Their findings revealed that pea protein isolate creates stronger gels than lupin protein isolate, with higher viscoelastic values, when testing gels made with 16% of protein. In addition, according to a study by Al-Ali et al. [51], lupin proteins form weaker gels because the proteins are highly compact and heat-stable, leading to a suppression of protein unfolding and S-S bond formation. However, it must be taken into account that protein gelling ability depends on the pH, ionic strength, heating and cooling conditions, and protein concentration [50].

LS MAD (5% of potato starch) and prototype 5% PPC showed very similar mechanical spectra, with no significant differences (*p* < 0.05) for G′ at 1 Hz, but LS MAD showed a significantly higher plateau modulus (G_N_^0^). It should be noted that potato starch and PPC do not have the same functionality since the former is a starch and the latter is a protein. Evaluating both prototypes, starch gelation occurred in the LS MAD, resulting in a softer and smoother texture to the taste compared to prototype 5% PPC, where protein gelation occurred, resulting in a less cohesive and gelatinous texture. This may be explained by the low protein concentration added in the 5% PPC prototype, which is insufficient to form a firmer structure.

The LS MAD prototype was the most similar to the MAD prototype, showing no significant differences (*p* < 0.05) in both G′ at 1 Hz and G_N_^0^ parameters. In addition, both MADs revealed no significant differences (*p* < 0.05) regarding the pH value (6.55 and 6.63, respectively). However, the color difference was slightly perceptible by the human eye, as the ΔE value was 7.82, since LS MAD had a darker and more intense pink color (L* = 71.2 ± 0.69, a* = 13.8 ± 0.88, b* = 13.6 ± 0.36) than the MAD prototype (Table 1).

Due to these results, and the lack of significant rheology differences (*p* < 0.05), the LS MAD was selected for texture in the replacements of E-number hydrocolloids by different clean-label texturizers. The best formulation sorted out was the one with a natural fiber (4%) (Optimized CL), which showed a homogeneous, juicy, and elastic texture, not brittle, doughy or gritty.

The E-number hydrocolloids used in this study were phosphates (E450–E452), carrageenan (E407), and konjac gum (E425). These traditional additives play a significant role in the functional quality of processed meat products. Phosphates serve as effective emulsifiers, enhancing water retention and texture, while also minimizing cooking losses. Carrageenan is commonly used to improve the texture and juiciness of meat products, and konjac gum contributes to moisture retention and fat reduction, supporting improved sensory properties and oxidative stability. These ingredients are widely used in the food industry for their consistent performance and technological efficiency, particularly in low-fat meat formulations [9,10].

Clean-label alternatives such as psyllium, pre-gelatinized banana starch, inulin, fructooligosaccharides (FOSs), and flours from cassava, rice, and quinoa offer promising functionalities, though with some limitations. Psyllium, for example, shows strong water-binding and gelling capabilities at low energy inputs, demonstrating similar rheological properties to xanthan gum [19,20,21]. Pre-gelatinized banana starch acts as a good bulking and thickening agent, with notable gelation potential at concentrations above 8% [22,23].

Inulin has limited thickening ability due to its small molecular size and low water-binding capacity, requiring concentrations of at least 15% to form stable gels. At moderate temperatures (60–70 °C), inulin gel hardness increases, but it weakens above 80 °C [24,25]. Fructooligosaccharides (FOSs) can mimic fat crystal structures, enhancing both taste and texture, though their use may lead to higher cooking losses in meat products [26,27].

Cassava, rice, and quinoa flours can form structured gels, with their performance influenced by temperature, starch concentration, and cooking time [28,29]. Although these clean-label ingredients may not fully match the functional reliability of traditional hydrocolloids, they present a viable natural alternative aligned with current consumer preferences for additive-free and minimally processed foods.

**Figure 3:**
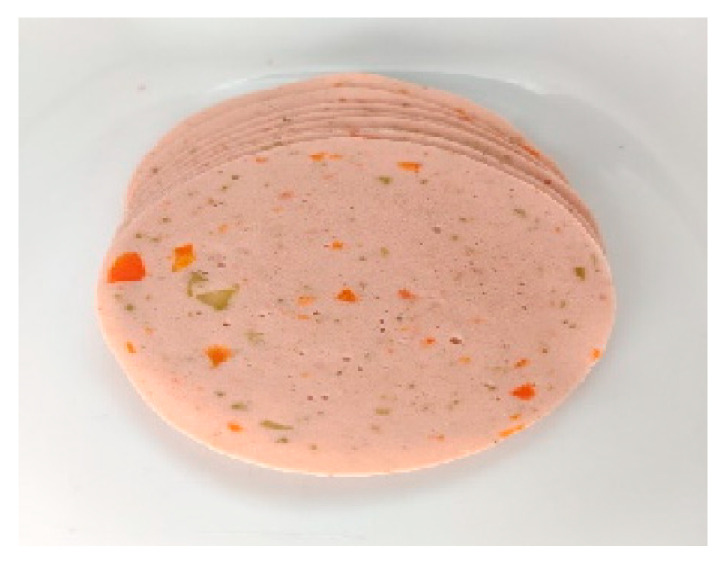
Pilot-scale product (CL MAD).

Once the desirable prototype was obtained at the laboratory scale, the scale-up of this product (CL MAD) (Figure 3) was carried out at pilot-scale facilities. To enable this transition, several adjustments were made to optimize the formulation for larger-scale production and to enhance the product’s sensory and technological characteristics, making it more comparable to commercially available MAD products. These adjustments included a slight change in the ingredient incorporation order to improve processing efficiency and product consistency, a reduction in color and water content to enhance visual appeal and texture, and the addition of vinegar powder as a clean-label preservative to improve shelf-life. Moreover, flavoring agents and vegetables were incorporated based on typical features observed in commercial MAD products.

The physicochemical, rheology and texture characteristics of the Optimized CL, the CL MAD, and the Target MAD are shown in Table 2. Regarding the TPA test results, when upscaling MAD formulation at pilot scale, a considerable improvement on its texture was observed, bringing it closer to the Target MAD sample. In relation to the texture measured, the Target MAD sample presented the highest adhesiveness and the CL MAD the highest cohesiveness. In addition, it could be noticed that the CL MAD presented higher firmness in relation to Optimized CL; however, it did not present significantly different (*p* > 0.05) values in relation to the Target MAD sample.

In a study, Carvalho et al. [52] developed a hybrid deli ham by partially replacing meat with plant-based proteins, using chickpea and red lentil flours combined with isolated potato protein. This formulation, containing equal proportions of animal and plant proteins, was compared to a conventional pork ham with respect to texture. The control sample demonstrated greater firmness and cohesiveness, while adhesiveness remained similar between both products. Similarly, Fan et al. [53] developed faba bean-based meat analogues incorporating brewers’ spent grain and observed that enzymatic treatment with transglutaminase further enhanced the texture, leading to noticeable increases in both hardness and cohesiveness.

According to Chen et al. [54], texture properties, including texture profile analysis, are important indicators for evaluating meat analogues and are related to consumer acceptance and purchasing behavior. Although progress has been made in recent years, research involving texture and flavor aspects will be fundamental to achieve better results.

Regarding color, the difference between the CL MAD and the Target MAD is slightly perceptible by the human eye (∆E = 7.57). This result is due to the lower values of the L* and a* color coordinates of the CL MAD, and a significantly higher b* coordinate, being a darker and less pink MAD. This difference may be attributed to multiple factors, including the omission of nitrite curing salts, the addition of vinegar powder, and the natural pigments introduced by the incorporated vegetables.

The CL MAD has the lowest pH value (6.19), since vinegar powder was added as a clean-label preservative alternative. In any case, all samples presented pH values around 6. pH values have a substantial impact on protein structure and functional properties, such as enhanced solubility, water absorption capacity, foaming capacity, and stability, as well as emulsifying activity and stability. In general, the highest protein solubility occurs at high acid and alkaline pH values and the lowest solubility at pH around 4.0 [3,55,56].

In addition, the Optimized CL and the CL MAD showed no differences in a_w_ (*p* < 0.05). However, the moisture content of the CL MAD presented a significantly (*p* < 0.05) higher value than that of the Optimized CL, where the increase in scale probably provided a greater water retention capacity, and the incorporation of frozen vegetables reduced the overall moisture.

**Figure 4:**
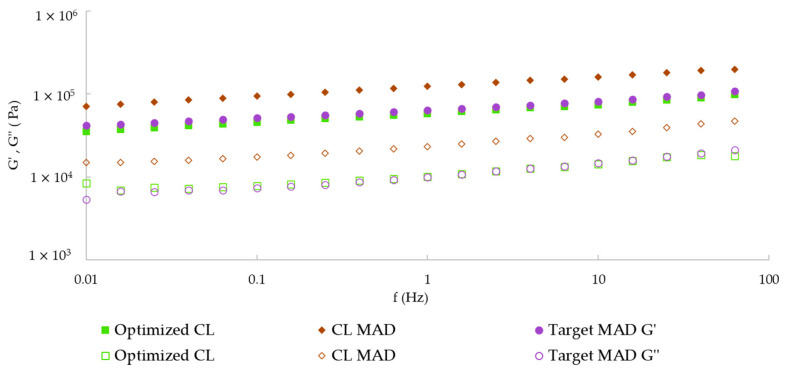
Modulus of conservation (elastic) G′ (solid symbols) and dissipation (viscous) G″ (open symbols) of the Optimized CL, CL MAD, and Target MAD.

According the rheological behavior of the Optimized CL, CL MAD, and Target MAD, all mechanical spectra revealed the same pattern, showing a structured weak gel behavior, with G′ over G″ and a slight frequency dependency (Figure 4). However, the CL MAD showed significantly (*p* < 0.05) higher values of both G′ at 1 Hz and G_N_^0^ (Table 2), revealing a stronger gel structure. This can be explained by various factors, such as (i) the different equipment at pilot scale, i.e., the pilot bowl cutter, the vacuum filler and sealing, etc.; (ii) different ingredient suppliers, particularly for the main thickening agents; (iii) adjustments made to the formulation, i.e., switching the order of incorporation of ingredients, reducing the water content, and adding more powdered ingredients (flavor agents and vinegar powder); among others. Furthermore, the added vegetables may have also interfered with some of the CL MAD rheology measurements.

Table 3 shows the nutritional information, where it can be noticed that the CL MAD has a protein content of 11 g/100 g, which represents 21% of the total energy value, meaning that the claim “High Protein” can be applied, as at least 20% of the energy value of the food must be provided by protein [57,58]. This claim is particularly important for people who follow a vegetarian diet, since an adequate protein content is crucial to avoid an insufficient protein intake, and it is essential to incorporate protein-rich foods in their diets [57].

Regarding the lipids, the CL MAD presented 15 g/100 g, which is in agreement with a study carried out by Locatelli et al. [59], who analyzed labels of 349 of plant-based meat analogue products and verified that the minimum and the maximum values presented a wide range (0.00 and 73.30 g/100 g, respectively). According Xie et al. [60], lipids play an important role in the generation of flavor compounds, for example, in plant-based meat analogue products. These also contribute to the juiciness and softness of the product, so the addition of vegetable oils is considered necessary.

Comparing the nutrition information of the CL MAD and the Target MAD, they show very similar results, which is very interesting, since the ingredients and percentages used were very distinct. The CL MAD has a higher protein and a lower fat content compared to the Target MAD, which is seen as very positive since it can be more appealing to the consumer.

**Figure 5:**
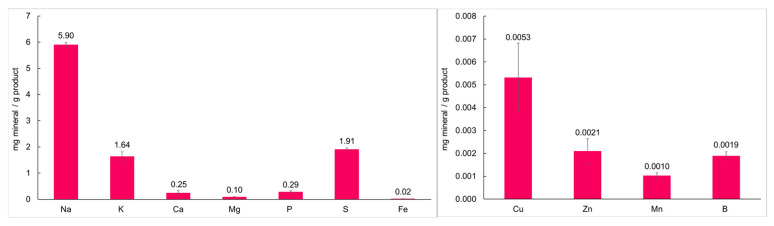
Mineral composition of the CL MAD.

With the growing consumption of plant-based products worldwide, it is also important to know their nutritional composition in terms of minerals, as they are considered essential elements, necessary for the normal metabolic functioning of the human body. Moreover, several researchers argue that plant-based products lack a number of micronutrients when compared to animal-derived meat [61,62]. Analyzing the mineral composition of the CL MAD (Figure 5), it can be seen that the CL MAD contains mainly sodium, sulfur, and potassium, as well as smaller amounts of calcium, magnesium, phosphorus, iron, copper, zinc, manganese, and boron.

The sodium content was a result of the added salt, which plays a crucial role in imparting flavor to food, preserving it, and regulating bodily functions. However, most people consume excessive amounts of salt. Therefore, it is necessary to adopt salt-reduction measures to improve people’s general level of health [63].

The presence of sulfur and potassium is most likely associated with the 10% of added vegetables, as carrots and broccoli are known to be rich sources of sulfur and sulfates [64]. Sulfur is an essential element required by all living organisms, as it is a fundamental component of proteins and various bio-organic compounds. Potassium, in turn, plays a crucial role in numerous cellular processes, such as maintaining intracellular fluid balance, enabling muscle contraction, transmitting nerve impulses, and helping to regulate blood pressure [65,66].

As for the other minerals, calcium plays a key role in bone strength and is vital for processes such as blood clotting, muscle contraction, and nerve transmission. Iron is essential for transporting oxygen, supporting cell division and gene regulation, and it binds to hemoglobin, giving blood its characteristic red color. Magnesium is crucial for maintaining cellular balance and proper organ function, contributing to processes such as oxidative phosphorylation, energy metabolism, glycolysis, and the synthesis of proteins and nucleic acids [62].

Yeo et al. [61] investigated the mineral content in plant-based meat analogues (PBMAs) and meat products, and the results showed that due to the extensive fortification of PBMAs, almost all of the PBMAs contained greater amounts of micronutrients compared to the animal meat counterparts. The PBMAs had significantly higher calcium, copper, iron, magnesium, manganese, and sodium than the meat products, while the meat products had significantly higher potassium levels than the PBMAs.

Youssef et al. [67] observed high Ca (95.43 mg/100 g) and Fe (7.72 mg/100 g) contents and lower Mn and Zn contents in burger soy tempeh, which may be due to the fermentation process. In another study, Higuera et al. [68] developed vegetable protein-based burgers formulated with soybean and pea proteins that had high levels of Ca, Fe, Mg, Mn, and Zn. The studies showed that the composition of protein-based burgers can be significantly influenced by the protein ingredients used in the formulation, as well as by other components, such as flavorings, colorings, stabilizers, and antioxidants, and by the type of processing used.

Regarding the overall liking evaluation of the six samples (the MAD prototype, the CL MAD, and the four commercial deli ham analogues), significant differences were found between samples (Table 4). The sample with a higher liking score was the Commercial 1 MAD (7.6 ± 1.4 out of 9 points), with no significant difference for the Commercial 3, Target MAD, and MAD prototypes. The liking results for the CL MAD presented a score of 6.1 ± 1.7 and showed a total of 80% positive responses, with no significant differences for the least preferred sample (Commercial 2).

**Figure 6:**
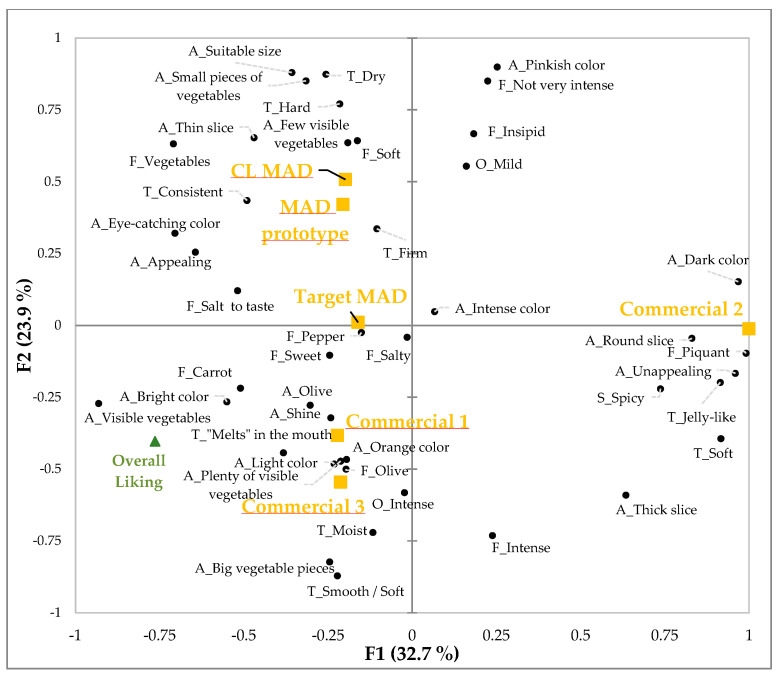
Projective mapping of sensory and visual attributes and consumer overall liking. The biplot displays the distribution of the MAD prototypes (CL MAD, MAD prototype) and commercial products (Target MAD, Commercial 1, 2, and 3) in the sensory space defined by the first two principal components (F1: 32.7%, F2: 23.9%), represented in yellow squares. Sensory and visual descriptors were projected as black dots (A—appearance; O—odor; T—texture; F—flavor), and the overall liking projection was plotted with a green triangle.

Figure 6 illustrates the first two dimensions of the MFA consensus, which account for 56.6% of the variability in the experimental data. The results reveal a strong correlation between Commercial 1 and 3, as well as Target MAD, with attributes such as bright and orange colors, visibility and an abundance of vegetables, pepper, sweet, salty, olive, and carrot flavors.

The CL MAD and MAD prototypes, aside from differences in liking, are perceived as having similar sensory characteristics, such as appealing and eye-catching colors, but with fewer visible vegetables and a firm texture, as well as mild vegetable flavors. The CL MAD seems to be more correlated with dry and hard texture than the MAD prototype.

The first dimension clearly separates the sample Commercial 2 from the remaining ones, and it is described as having a dark color, unappealing, with a thick and round appearance, jelly-like and soft texture, and piquant flavor.

Through this analysis, it was possible to understand that the liking of the sample is related to the presence of visible vegetables, firm but hard and dry texture, and a vegetable taste. Thus, two key factors for improving the liking of these products were identified: (i) the appearance (large irregular pieces of vegetables) and (ii) the intensity of the vegetable flavor. In terms of texture, the CL MAD can be improved in terms of dryness and hardness. In the future, further improvements can be made to enhance the overall appeal of the CL MAD. Small visible pieces of vegetables can be added in a larger size. Regarding the mild flavor, this can be overcome, if necessary, by using higher intensity flavoring agents, for example.

## 4. Conclusions

This study successfully developed a clean-label, meat-free alternative to deli ham (CL MAD), using natural and minimally processed ingredients. Through successive formulation trials and optimization at both laboratory and pilot scales, significant improvements were achieved in the physicochemical, textural, and rheological properties of the product. The final prototype demonstrated mechanical and nutritional characteristics comparable to a commercial benchmark, with the added benefit of eliminating synthetic additives. Moreover, the CL MAD achieved a high protein content, reduced fat levels, and favorable consumer acceptance, highlighting its potential as a sustainable and health-conscious alternative in the growing plant-based market. Furthermore, due to the rise of the meat-like plant-based products market, it is important to continue studying and developing more new products to meet consumer expectations.

## Figures and Tables

**Table 1:** Characteristics of the MAD prototype and the Target MAD.

Analysis	Parameters	MAD Prototype	Target MAD
Color	L*	76.37 ± 0.454 ^a^	76.00 ± 0.261 ^a^
	a*	8.41 ± 0.183 ^a^	8.93 ± 0.152 ^a^
	b*	15.89 ± 0.177 ^a^	10.86 ± 0.277 ^b^
Physicochemical characteristics	pH	6.55 ± 0.025 ^a^	6.29 ± 0.060 ^b^
	a_w_	0.96 ± 0.001 ^b^	0.97 ± 0.001 ^a^
	Moisture (%)	64.37 ± 0.599 ^a^	64.87 ± 0.469 ^a^
	Ash (%)	3.17 ± 0.296 ^a^	2.39 ± 0.357 ^b^
Rheology	G_N_^0^ (kPa)	31.32 ± 5.371 ^a^	43.83 ± 2.929 ^a^
	G′ at 1 Hz (kPa)	40.47 ± 4.627 ^a^	62.03 ± 3.648 ^a^
Texture	Firmness (N)	5.20 ± 0.290 ^b^	12.3 ± 0.360 ^a^
	Adhesiveness (-N/s)	1.08 ± 0.319 ^a^	1.04 ± 0.128 ^a^
	Cohesiveness	0.47 ± 0.053 ^b^	0.58 ± 0.013 ^a^

Results with different letters in the same row show significant differences (*p* < 0.05) for each parameter.

**Table 2:** Characteristics of the Optimized CL, CL MAD, and Target MAD products.

Analysis	Parameters	Optimized CL	CL MAD	Target MAD
Color	ΔE to Target MAD	14.2	7.57	-
Physicochemical characteristics	pH	6.64 ± 0.006 ^a^	6.19 ± 0.017 ^c^	6.29 ± 0.060 ^b^
	a_w_	0.95 ± 0.002 ^b^	0.93 ± 0.007 ^b^	0.97 ± 0.002 ^a^
	Moisture (%)	63.79 ± 0.295 ^b^	65.19 ± 0.409 ^a^	64.87 ± 0.469 ^a^
	Ash (%)	1.76 ± 0.046 ^b^	1.58 ± 0.087 ^b^	2.39 ± 0.357 ^a^
Rheology	G′ at 1 Hz (kPa)	58.50 ± 6.213 ^b^	122.97 ± 1.721 ^a^	62.03 ± 3.648 ^b^
	G_N_^0^ (kPa)	43.79 ± 5.785 ^b^	101.13 ± 4.428 ^a^	43.83 ± 2.929 ^b^
Texture	Firmness (N)	7.20 ± 0.710 ^b^	12.00 ± 0.660 ^a^	12.30 ± 0.360 ^a^
	Adhesiveness (-N/s)	0.07 ± 0.045 ^c^	0.52 ± 0.302 ^b^	1.04 ± 0.128 ^a^
	Cohesiveness	0.79 ± 0.015 ^a^	0.68 ± 0.020 ^b^	0.58 ± 0.013 ^c^

Results with different letters in the same row showed significant differences (*p* < 0.05) for each parameter.

**Table 3:** Nutritional information of the CL MAD and the Target MAD (wet basis).

Nutritional Information	Content (Uni.) Per 100 g	
	CL MAD	Target MAD *
Energy (kcal/kJ)	207.6/868.7	203.0/843.0
Lipids (g)	15.0	16.0
Carbohydrate (g)	7.3	5.1
Protein (g)	11.0	8.6

* Nutritional data based on label information.

**Table 4:** Mean overall liking scores and percentage of positive answers for the six samples evaluated in the sensory analysis.

Samples	Overall Liking ^1^	% Positive Answers
MAD prototype	7.3 ± 1.3 ^a^	94
CL MAD	6.1 ± 1.7 ^b^	80
Target MAD	7.0 ± 1.8 ^a^	86
Commercial 1	7.6 ± 1.4 ^a^	94
Commercial 2	5.6 ± 2.0 ^b^	63
Commercial 3	7.2 ± 1.3 ^a^	95

^1^ Overall liking ranging between 1—dislike extremely and 9—like extremely. ^a,b^—homogeneous groups according to the Wilcoxon non-parametric test, at a 95% confidence level.

## Data Availability

The original contributions presented in this study are included in the article. Further inquiries can be directed to the corresponding author(s).
